# Неклассическая форма липоидной гиперплазии надпочечников: описание клинических случаев

**DOI:** 10.14341/probl13589

**Published:** 2026-01-18

**Authors:** И. Г. Сичинава, Е. С. Демина, Е. М. Шарибжанова, Л. С. Созаева, Е. Е. Петряйкина, А. Н. Тюльпаков

**Affiliations:** Российский национальный исследовательский медицинский университет им. Н.И. ПироговаРоссия; Pirogov Russian National Research Medical UniversityRussian Federation; Российский национальный исследовательский медицинский университет им. Н.И. Пирогова; Российская детская клиническая больницаРоссия; Pirogov Russian National Research Medical University; Russian Children’s Clinical HospitalRussian Federation; Российская детская клиническая больницаРоссия; Russian Children’s Clinical HospitalRussian Federation; Национальный медицинский исследовательский центр эндокринологии им. акад. И.И. ДедоваРоссия; Endocrinology Research CenterRussian Federation; Российская детская клиническая больница; Медико-генетический научный центр имени академика Н.П. БочковаРоссия; Russian Children’s Clinical Hospital; Research Center for Medical GeneticsRussian Federation

**Keywords:** липоидная гиперплазия надпочечников, неклассическая форма липоидной гиперплазии надпочечников, мутации в гене STAR, дефект белка StAR, стероидогенез, lipoid adrenal hyperplasia, non-classical lipoid adrenal hyperplasia, STAR gene mutations, StAR protein defect, steroidogenesis

## Abstract

Липоидная гиперплазия надпочечников (ЛГН) является редкой тяжелой формой врожденной дисфункции коры надпочечников, в основе которой лежат мутации в гене STAR (8p11.2), кодирующем транспортный белок StAR. Дефект белка StAR приводит к тотальному нарушению стероидогенеза в надпочечниках и гонадах. Деление на классическую форму заболевания, при которой нарушается весь стероидогенез, и неклассическую, при которой обычно нарушается только стероидогенез надпочечников, — общепризнанная классификация ЛГН. Представлены результаты двух наблюдений пациентов, у которых клинико-лабораторный спектр указывал на неклассическую форму ЛГН. Для обоих пациентов с кариотипом 46,XY были характерны поздняя (в 5 лет и в 3 года) манифестация клинических симптомов и нормальное строение мужских гениталий. При молекулярно-генетическом исследовании у одного пациента была выявлена гомозиготная мутация p.R188C, а у другого — компаунд-гетерозиготные мутации p.R188C и p.R272H. Генетическое тестирование STAR позволило диагностировать неклассическую форму ЛГН, о которой надо помнить при дифференциальной диагностике гипокортицизма.

## Abstract (en)

Lipoid adrenal hyperplasia (LAH) is a rare severe form of congenital adrenal hyperplasia, which is caused by mutations in the STAR gene (8p11.2), encoding the transport protein StAR. The defect of the StAR protein leads to a total disruption of adrenal and gonadal steroidogenesis. The division into the classic form of the disease, in which all steroidogenesis is disrupted, and the non-classic, in which only adrenal steroidogenesis is usually disrupted, is a generally accepted classification of LAH. We report two cases of patients with clinical and laboratory data indicating a non-classic LAH. Both patients with 46,XY karyotype presented late (at 5 and 3 years) manifestation of clinical symptoms and had normal male external genitalia. Homozygous mutation p.R188C in one patient and compound heterozygous mutations p.R188C and p.R272H in the other were identified by molecular genetic assay. STAR genetic testing allowed to diagnose non-classic LAH in differential diagnosis of hypocorticism.

## АКТУАЛЬНОСТЬ

Липоидная гиперплазия надпочечников (ЛГН) является редкой тяжелой формой врожденной дисфункции коры надпочечников. В основе ЛГН лежат мутации в гене STAR (8p11.2), который кодирует транспортный белок StAR (стероидогенный острый регулятор) [1]. Белок StAR отвечает за перемещение холестерина с наружной на внутреннюю мембрану митохондрии, где и запускается процесс ферментативного превращения холестерина в стероидные гормоны. Дефект белка StAR приводит к тотальному нарушению стероидогенеза в надпочечниках и гонадах [2].

Клиника ЛГН манифестирует с первых дней жизни и, как правило, характеризуется тяжелой первичной надпочечниковой (глюко- и минералокортикоидной) недостаточностью (ПНН) и инверсией фенотипического пола у пациентов с кариотипом 46,XY. Однако в 2006 г. Baker с соавт. [3] описали 3 клинических случая с более легкой формой ЛГН, проявляющейся клиникой изолированной надпочечниковой недостаточности с дебютом в более старшем возрасте (в 2–4 года) и нормальным развитием мужских гениталий у пациентов с кариотипом 46,XY. По мнению авторов, эта форма заболевания, которую они назвали «неклассическая липоидная гиперплазия надпочечников», иллюстрирует, что клинические проявления нарушений стероидогенеза могут отличаться от их классических описаний и определяют новую неаутоиммунную болезнь Аддисона. В настоящее время деление на классическую и неклассическую формы заболевания — общепризнанная классификация ЛГН.

Редкость ЛГН исключает возможность проведения масштабных эпидемиологических и клинических исследований. Данные о распространенности ЛГН ограничены даже в различных регионах Восточной Азии, где это заболевание встречается чаще. На сегодняшний день, по данным различных источников, в мире зарегистрировано около 250 пациентов с ЛГН, вызванных 120 разными вариантами в гене STAR [4][5].

В отечественной литературе описание случаев ЛГН встречается лишь с 2006 г., когда впервые были представлены данные о трех пациентах, у которых диагноз классической формы был верифицирован на основании молекулярно-генетического обследования — мутации в гене STAR [6].

В настоящей публикации представлены результаты двух наблюдений пациентов с ЛГН, у которых клинико-лабораторный спектр нарушений указывает на неклассическую форму заболевания.

## ОПИСАНИЕ СЛУЧАЯ

Пациент №1, 17 лет 10 мес. Мальчик от 1-й беременности, первых срочных самостоятельных родов. Масса тела при рождении — 3000 г, длина тела неизвестна. Семейный анамнез неизвестен (воспитывается в приемной семье).

С раннего возраста обращали на себя внимание плохая прибавка в росте и весе, слабость, утомляемость, двусторонний крипторхизм. С 5 лет стали отмечаться приступы рвоты, жидкого стула, расцениваемые как кишечная инфекция.

В возрасте 6 лет заподозрена первичная надпочечниковая недостаточность (ПНН), назначена терапия гидрокортизоном и флудрокортизоном ацетатом. В 9 лет 7 мес и 10 лет перенес операции по поводу двустороннего крипторхизма.

При обследовании в возрасте 10 лет 3 мес — на заместительной гормональной терапии глюкокортикоидами (гидрокортизон 7,7 мг/м²/сут) и минералокортикоидами (флудрокортизона ацетат 50 мкг/сут) АКТГ 1947 пг/мл (норма 7,2–63,3), активность ренина плазмы (АРП) 38,5 нг/мл/час (норма 0,5–3,3). Доза гидрокортизона увеличена до 12,8 мг/м²/сут, флудрокортизона ацетата — до 75 мкг/сутки.

Для уточнения этиологии гипокортицизма проведено молекулярно-генетическое исследование. Учитывая сочетание надпочечниковой недостаточности и крипторхизма в качестве наиболее вероятного диагноза рассматривалась врожденная гипоплазия надпочечников, однако при секвенировании методом Сэнгера гена NR0B1 изменений выявлено не было. При проведении массового параллельного секвенирования «Надпочечниковая недостаточность и электролитные нарушения» (38 генов) был обнаружен гомозиготный вариант в гене STAR (NM_000349): с.562С>T:p. R188C.

При контрольном обследовании в возрасте 17 лет 10 мес: рост — 161,0 см (SDS=-2,0), вес — 55,5 кг (SDS ИМТ=-0,1), отмечается гиперпигментация кожи, более выраженная в области крупных суставов, ЧСС 72 уд/мин, АД 110/70 мм рт.ст. Половые органы сформированы правильно, по мужскому типу, половое развитие по Таннеру G4P5, объем яичек 12–15 мл.

Данные лабораторного обследования: натрий — 138,8 ммоль/л (норма 136–145), калий — 4,0 ммоль/л (норма 3,5–5,1), мочевина — 3,0 ммоль/л (норма 3–7,5), фосфор — 1,2 ммоль/л (норма 0,95–1,6), кальций общий — 2,4 ммоль/л (норма 2,1–2,6), ренин (прямой) — 9,6 мЕд/л (норма 2,8–39,9), АКТГ (утро) — 188,9 пг/мл (норма 7,2–63,3), ФСГ — 14,7 Ед/л (норма 1,6–9,7), ЛГ 16,8 Ед/л (норма 2,5–11), тестостерон — 12,1 нмоль/л (норма 11–28,2).

УЗИ органов мошонки: яички в мошонке, правое яичко: 4,21х2,51х1,91 см, левое яичко 3,94х1,94х2,19 см, киста головки придатка левого яичка, оболочечные кальцинаты 0,7–2,2 мм с двух сторон.

Пациент №2, 13 лет 5 мес. Ребенок от первой нормально протекавшей беременности, срочных родов путем кесарева сечения. При рождении масса тела — 3800 г, длина тела — 56 см. Оценка по шкале Апгар 7–8 баллов. Наружные гениталии развиты правильно, по мужскому типу, тестикулы в мошонке. Развитие на первом году жизни без особенностей. Из анамнеза известно, что брак неблизкородственный.

Наследственный анамнез отягощен: у матери — сахарный диабет 1 типа (СД1), аутоиммунный тиреоидит; у бабушки по линии матери — сахарный диабет 2 типа (СД2), диффузный токсический зоб.

Со слов мамы, у ребенка в возрасте с 3 до 6 лет отмечались периодические эпизоды резкой слабости, вялости, рвоты на фоне повышения температуры тела, купируемые внутривенным введением глюкозы в условиях стационара. Документация о ранее проведенных обследованиях отсутствует.

Причиной обращения в возрасте 8 лет были жалобы родителей на невнимательность, нарушение памяти и поведения ребенка. При осмотре отмечена гиперпигментация кожных покровов, слизистых ротовой полости и мест трения. По результатам проведенного обследования [ АКТГ — 4956,0 пг/мл (норма 7,2–63); кортизол — 1,8 мкг/мл (норма 6,2–19,4); 17-ОН-прогестерон — 0,26 нг/мл (норма 0,2–0,8); глюкоза — 4,1 ммоль/л (норма 3,3–5,6)] был установлен диагноз первичной надпочечниковой недостаточности. Данных за дефицит минералокортикоидов не было получено [ натрий — 140,7 ммоль/л (норма 132–145); калий — 4,5 ммоль/л (норма 3,5–5,1); альдостерон — 75,3 пмоль/л (норма 48,7–653,7); ренин прямой — 24,95 мкМЕ/мл (норма 2,8–39,9)]. При проведении КТ надпочечников патологии не было выявлено. Назначена заместительная гормональная терапия глюкокортикоидами (гидрокортизон 10 мг/сут).

В возрасте 8 лет 10 мес были проведены дополнительные исследования с целью дифференциальной диагностики между различными вариантами первичной надпочечниковой недостаточности: исключены Х-сцепленная адренолейкодистрофия (концентрация очень длинноцепочечных жирных кислот (ОДЦЖК) и соотношение концентраций кислот в пределах нормы) и аутоиммунный полигландулярный синдром 1 типа (нет повышения антител к интерферону-омега), изолированная аутоиммунная болезнь Аддисона (нет повышения антител к 21-гидроксилазе). При проведении массового параллельного секвенирования панели генов «Надпочечниковая недостаточность и электролитные нарушения» (38 генов) выявлено 2 гетерозиготных варианта в гене STAR (NM_000349): с.562С>T:p. R188C и с.815G>A:p. R272H. При дополнительном обследовании матери ребенка в гене STAR был обнаружен только один из двух указанных выше вариантов (p.R188C), что с большой вероятностью свидетельствовало в пользу компаунд-гетерозиготности вариантов p.R188C и p.R272H у пробанда. Отец пациента для обследования не был доступен.

Заместительная терапия глюкокортикоидами продолжена в прежней дозе (гидрокортизон 10 мг/сутки; 10,2 мг/м²/сутки) с рекомендациями по коррекции доз. Необходимости в проведении терапии минералокортикоидами по результатам биохимического анализа крови не было [ калий — 4,0 ммоль/л (норма 3,5–5,1); натрий — 141 ммоль/л (норма 136–145)].

В возрасте 10 лет 6 мес рост — 135 см (SDS роста=-0,69), вес — 41 кг (SDS ИМТ=+2,07). При осмотре обращала на себя внимание умеренная гиперпигментация кожных покровов. Половое развитие по Таннеру G3P3, тестикулы в мошонке, объем 10–12 мл. Костный возраст (TW20): 13,5 лет. ЛГ — 3,5 МЕ/л (норма 0,6–6,3); ФСГ — 0,92 МЕ/л (норма 1,2–6,8)], тестостерон — 8,18 нмоль/л (норма 0,5–9,7)].

Продолжена терапия гидрокортизоном (15 мг/сут; 12,3 мг/м²/сут) на фоне которой при гормональном обследовании и клинически отмечалось удовлетворительное течение заболевания. Учитывая нормальные показатели минерального обмена [ калий — 3,9 ммоль/л (норма 3,4–4,7), натрий — 141 ммоль/л (норма 136–145), рениновая активность — 3,4 нг/мл/ч] от терапии минералокортикоидами по-прежнему решено было воздержаться.

В возрасте 11 лет 1 мес при УЗИ мошонки выявлены эхо-признаки кальцинатов в обоих тестикулах: в правом — два рядом расположенные гиперэхогенных включения 4х2 мм и 2х1,7 мм без акустической тени, головка придатка 9x7 мм с жидкостным образованием 3х2,8 мм с толстым гиперэхогенным контуром; в левом — гиперэхогенное включение 5х3 мм без акустической тени и гиперэхогенное включение 3,3х1,3 мм с акустической тенью, головка придатка 8x7 мм. В процессе динамического наблюдения в течение 2 лет при УЗИ не выявлено отрицательной динамики, эхопризнаки кальцинатов описываются только в левом тестикуле.

При контрольном обследовании в возрасте 13 лет 5 мес на фоне проводимой заместительной терапии глюкокортикоидами (гидрокортизон 15 мг/сут; 10,3 мг/м²/сут): рост 152 см (+8 см за 1 год, SDS роста=-0,55); вес 50 кг (SDS ИМТ=+1,25). Костный возраст (TW20): 14 лет. Кожные покровы смуглые, гиперпигментация типичных мест (локтевые сгибы, область коленных суставов, кайма губ, подмышечные ямки, ареолы сосков, наружные половые органы). Половое развитие по Таннеру G4-5P4, тестикулы в мошонке, объем 20 мл. АД 98/66 мм рт.ст. По данным лабораторного обследования: ЛГ — 5,9 МЕ/л (норма 0,6–7,8); ФСГ — 0,6 МЕ/л (норма 1,5–6,8), тестостерон — 15,1 нмоль/л (норма 9,2–27,8) калий — 4,11 ммоль/л (норма 3,4–4,7), натрий — 140 ммоль/л (норма 136–145), рениновая активность плазмы — 0,4 нг/мл/ч (норма 0,5–3,3).

## ОБСУЖДЕНИЕ

Критерии неклассической ЛГН включают поздний возраст начала заболевания и развитие наружных гениталий соответственно хромосомному полу [2]. Согласно данным литературы, возраст манифестации (постановки диагноза) при неклассической форме ЛГН в большинстве случаев варьирует от 2 до 4 лет (табл. 1). Между тем описаны случаи, когда диагноз устанавливался уже у взрослых. Так, Metherell с соавт. [7] обследовали семью, где гомозиготный вариант p.R188C в гене STAR был выявлен у девочки с манифестацией ПНН в 2 года, и такая же гомозиготная мутация была обнаружена у дяди пробанда, но при этом у него до 58 лет не был диагностирован гипокортицизм и не проводилось лечение.

**Table:** Таблица 1. Характеристика пациентов с неклассической формой ЛГН (мутации p.R188C и p.R272H) по данным литературы и собственных наблюдений Примечание: М — мужской фенотип, Ж — женский фенотип, С — смешанный фенотип, Г — гипоспадия, МП — микропенис, 2стК — двусторонний крипторхизм, 1стК — односторонний крипторхизм.

Кариотип	Генотип	Возраст дебюта/ постановки диагноза	Фенотип (наружные гениталии)	Источник литературы
46,XY	p.[ R188C];[ R188C]	2,2 года	М	Baker et al., 2006 [3]
46,XY	p.[ R188C];[ R188C]	2,8 года	М	Baker et al., 2006 [3]
46,XX	p.[ R188C];[ R188C]	2 года	Ж	Metherell et al., 2009 [7]
46,XY	p.[ R188C];[ R188C]	Не сообщается	М	Metherell et al., 2009 [7]
46,XY	p.[ R188C];[ R188C]	58 лет	М	Metherell et al., 2009 [7]
46,XY	p.[ R188C];[ R188C]	6 лет	М	Metherell et al., 2009 [7]
46,XY	p.[ R188C];[ R188C]	Не сообщается	М	Metherell et al., 2009 [7]
46,XX	p.[ R188C];[ R188C]	1,5 года	Ж	Metherell et al., 2009 [7]
46,XY	p.[ R188C];[ R188C]	<1 года	М, 2стК	Metherell et al., 2009 [7]
46,XY	p.[ R188C];[ R188C]	<1 года	М	Metherell et al., 2009 [7]
46,XY	p.[ R188C];[ R188C]	3 года	М, Г	Metherell et al., 2009 [7]
46,XY	p.[ R188C];[ R188C]	4 года	М, Г	Sahakitrungruang et al., 2010 [8]
46,XY	p.[ R188C];[ W250X]	3,5 мес	С (Г, МП, 2стК)	Sahakitrungruang et al., 2010 [8]
46,XX	p.[ R188C];[ R188C]	3 года	Ж	Tsai et al., 2016 [9]
46,XY	p.[ R188C];[ R188C]	6 лет	М	Tsai et al., 2016 [9]
46,XY	p.[ R188C];[ R188C]	9 лет	М	Tsai et al., 2016 [9]
46,XY	p.[ R188C];[ R188C]	4 года	М	Tsai et al., 2016 [9]
46,XX	p.[ R188C];[ R188C]	17 лет	Ж	Tsai et al., 2016 [9]
46,XY	p.[ R272H];[ Q258X]	Не сообщается	М	Kang et al., 2017 [10]
46,XX	p.[ R272H];[ Q258X]	2,4 лет	Ж	Kang et al., 2017 [10]
46,XX	p.[ R188C];[ R188C]	14 мес	Ж	Burget et al., 2018 [11]
46,XY	p.[ R188C];[ R188C]	2 года	М	Burget et al., 2018 [11]
46,XY	p.[ R272H];[ Q258X]	5 лет	М	Ishii T. et al., 2019 [12]
46,XY	p.[ R272H];[ Q258X]	4 года	М, 1стК	Ishii T. et al., 2019 [12]
46,XY	p.[ R272H];[ Q258X]	1,5 года	М	Liang X. et al., 2021 [13]
46,XY	p.[ R188C];[ Q258X]	1,25 года	М, МП	Lu W. et al., 2022 [14]
46,XY	p.[ R188C];[ R188C]	5 лет	М, 2стК	собственные данные
46,XY	p.[ R188C];[ R272H]	3 года	М	собственные данные

Что касается строения наружных гениталий у пациентов с кариотипом 46,XY и неклассической формой ЛГН, то у большинства оно правильное мужское, однако также описаны случаи с неполной маскулинизацией. По обзорным данным Lu с соавт. [14], гипоспадии разной степени выраженности выявляются у 21,5% таких пациентов. Частично фенотипы могут быть объяснены молекулярными дефектами, затрагивающими функцию белка StAR, однако различное строение наружных гениталий отмечается и у пациентов с идентичными генотипами (табл. 1).

В представленных нами клинических случаях отмечалась поздняя манифестация надпочечниковой недостаточности (с 5 лет и с 3 лет жизни соответственно), которая проявлялась у обоих пациентов неспецифическими признаками (периодические приступы слабости, рвоты) и прогрессировала постепенно, при этом заместительная гормональная терапия была назначена уже в возрасте старше 6 лет. Что касается компонентов гипокортицизма, то у пациента №1 данные гормонального обследования в возрасте 10 лет 3 мес четко указывали на сочетанный дефицит глюко- и минералокортикоидов, тогда как у пациента №2 гормональные показатели в большей мере свидетельствовали в пользу изолированного дефицита глюкокортикоидов. У обоих пациентов наружные половые органы были сформированы по мужскому типу, однако у пациента №1 имел место двусторонний паховый крипторхизм, что может свидетельствовать об относительном дефиците андрогенов на поздней стадии формирования половой системы.

У обоих пациентов отмечен самостоятельный старт пубертата. На спонтанное начало полового созревания и отсутствие необходимости в заместительной терапии половыми гормонами даже в более позднем возрасте у пациентов с неклассической формой отмечено при проведении многоцентрового поперечного когортного исследования пациентов с ЛГН в Японии [15]. Считается, что способность вырабатывать тестостерон может быть сохранена у большинства мужчин с неклассической ЛГН и позволяет развить мужские половые органы, достичь полного пубертатного развития и даже вызвать созревание репродуктивных клеток, несмотря на накопление липидов в клетках Лейдига [12].

По данным большинства исследователей, последствия «неклассических» мутаций на половое развитие пациентов невозможно оценить в детстве, требуется долгосрочное наблюдение в период полового созревания и взрослой жизни. Помимо этого, редкость неклассической формы ЛГН не позволяет до конца прояснить долгосрочные результаты функции яичек [3][12]. Именно динамическое наблюдение при УЗИ органов мошонки позволило у обоих пациентов выявить кальцинаты в тестикулах. Единичный случай выявления микрокальцификатов в одном из яичек был описан у мужчины в возрасте 31 года с неклассической ЛГН, у которого в анамнезе был перерыв в приеме глюкокортикоидной терапии в период с 17 до 30 лет [12]. Значимость выявления кальцинатов у описанных нами пациентов пока оценить сложно. У пациента №1 тестикулы длительно находились в состоянии крипторхизма, что могло иметь повреждающий эффект на ткань яичка, прежде всего семенных канальцев; у него же в пубертатном периоде отмечены высокие значения ФСГ.

Визуализационные исследования надпочечников (у пациента №2 изменений при КТ надпочечников не было выявлено), по мнению авторов, также не позволяют четко различать классическую и неклассическую форму ЛГН [15].

Как было указано выше, при молекулярно-генетическом исследовании у пациента №1 была выявлена гомозиготная мутация p.R188C, а у пациента №2 — компаунд-гетерозиготные мутации p.R188C и p.R272H. Оба варианта являются патогенными, о чем свидетельствуют предшествующие описания пациентов с подобными изменениями (табл. 1) и функциональные данные [14].

Именно на мутацию с.562С>T:p.R188C указывают как на наиболее распространенный вариант в гене STAR, ассоциированный с неклассической ЛГН, частота которого достигает 37,2% от общего числа мутантных аллелей и может отражать, по мнению авторов, эффект основателя [3][14]. Вторая гетерозиготная мутация p.R272H, выявленная у пациента №2, описана в работах корейских, японских и китайских ученых. Так Kang E. и соавт. [10] при исследовании спектра мутаций гена STAR у 45 корейских пациентов с ЛГН данная мутация была выявлена в составе компаунд-гетерозиготной мутации p.Q258X и p.R272H у одного генетического мужчины с правильными мужскими гениталиями и одной генетической женщины с дебютом ПНН без потери соли в возрасте 2,4 года. С аналогичной компаунд-гетерозиготной мутацией, а именно c.815G>A:p. R272H и c.772C>T:p. Q258X, Ishii T. и соавт. [12] описывают двух пациентов с ЛГН с полной вирилизацией наружных гениталий и спонтанным пубертатом, а китайские коллеги Liang X. и соавт. [13] представляют наблюдение за пациентом 1,5 лет с кариотипом 46,XY и полной вирилизацией наружных гениталий.

Мутации p.R188C и p.R272H являются вариантами с частичной потерей функции белка и коррелируют с более легким течением, поздним дебютом и вирилизирующим эффектом у пациентов с кариотипом 46,XY [3][14][15]. Функциональные анализы показали, что p.R272H сохраняет 35% активности StAR дикого типа in vitro, а p.R188C — от 12,8 до 13,6%. Таким образом, позднее начало заболевания и мужской генитальный фенотип у представленных нами пациентов могут быть связаны остаточной активностью StAR при выявленных мутациях.

## ЗАКЛЮЧЕНИЕ

Клинический фенотип пациентов с мутациями STAR варьирует и, как показали проведенные исследования, может быть сгруппирован в классическую ЛГН, при которой нарушается весь стероидогенез, и неклассическую ЛГН, при которой обычно нарушается только стероидогенез надпочечников. При стертых вариантах ЛГН может проявляться поздней манифестацией, а также нормальным мужским строением половых органов и сохраненной фертильностью, или сопровождаться легкими вариантами нарушения формирования пола (головчатой гипоспадией или крипторхизмом).

Учитывая разнообразие клинических фенотипов неклассической формы заболевания необходимы помимо тщательного клинико-лабораторного обследования, проведение молекулярно-генетического исследования. Генетическое тестирование STAR позволяет подтвердить неклассическую форму ЛГН, имеющую важное значение с точки зрения дифференциальной диагностики гипокортицизма.

## ДОПОЛНИТЕЛЬНАЯ ИНФОРМАЦИЯ

Источники финансирования. Работа выполнена по инициативе авторов без привлечения финансирования.

Конфликт интересов. Авторы декларируют отсутствие явных и потенциальных конфликтов интересов, связанных с содержанием настоящей статьи.

Участие авторов. Все авторы одобрили финальную версию статьи перед публикацией, выразили согласие нести ответственность за все аспекты работы, подразумевающую надлежащее изучение и решение вопросов, связанных с точностью или добросовестностью любой части работы.

Согласие пациента. Законные представители пациентов добровольно подписали информированное согласие на публикацию персональной медицинской информации в обезличенной форме в журнале «Проблемы эндокринологии».
